# Mobile Compensatory Mutations Promote Plasmid Survival

**DOI:** 10.1128/mSystems.00186-18

**Published:** 2019-01-15

**Authors:** Martin Zwanzig, Ellie Harrison, Michael A. Brockhurst, James P. J. Hall, Thomas U. Berendonk, Uta Berger

**Affiliations:** a Department of Forest Sciences, Institute of Forest Growth and Forest Computer Science, Technische Universität Dresden, Tharandt, Germany; b Department of Animal and Plant Sciences, University of Sheffield, Sheffield, United Kingdom; c Department of Evolution, Ecology and Behaviour, Institute of Integrative Biology, University of Liverpool, Liverpool, United Kingdom; d Department of Hydro Sciences, Institute of Hydrobiology, Technische Universität Dresden, Dresden, Germany; University of California, San Diego

**Keywords:** compensatory evolution, chromosomal mutation, plasmid mutation, plasmid persistence, fitness costs, cost compensation, antibiotic resistance, mathematical modeling, conjugation, horizontal gene transfer

## Abstract

The global dissemination of plasmids encoding antibiotic resistance represents an urgent issue for human health and society. While the fitness costs for host cells associated with plasmid acquisition are expected to limit plasmid dissemination in the absence of positive selection of plasmid traits, compensatory evolution can reduce this burden. Experimental data suggest that compensatory mutations can be located on either the chromosome or the plasmid, and these are likely to have contrasting effects on plasmid dynamics. Whereas chromosomal mutations are inherited vertically through bacterial fission, plasmid mutations can be inherited both vertically and horizontally and potentially reduce the initial cost of the plasmid in new host cells. Here we show using mathematical models and simulations that the dynamics of plasmids depends critically on the genomic location of the compensatory mutation. We demonstrate that plasmid-located compensatory evolution is better at enhancing plasmid persistence, even when its effects are smaller than those provided by chromosomal compensation. Moreover, either type of compensatory evolution facilitates the survival of resistance plasmids at low drug concentrations. These insights contribute to an improved understanding of the conditions and mechanisms driving the spread and the evolution of antibiotic resistance plasmids.

**IMPORTANCE** Understanding the evolutionary forces that maintain antibiotic resistance genes in a population, especially when antibiotics are not used, is an important problem for human health and society. The most common platform for the dissemination of antibiotic resistance genes is conjugative plasmids. Experimental studies showed that mutations located on the plasmid or the bacterial chromosome can reduce the costs plasmids impose on their hosts, resulting in antibiotic resistance plasmids being maintained even in the absence of antibiotics. While chromosomal mutations are only vertically inherited by the daughter cells, plasmid mutations are also provided to bacteria that acquire the plasmid through conjugation. Here we demonstrate how the mode of inheritance of a compensatory mutation crucially influences the ability of plasmids to spread and persist in a bacterial population.

## Abstract (precis)

Understanding the evolutionary forces that maintain antibiotic resistance genes in a population, especially when antibiotics are not used, is an important problem for human health and society. The most common platform for the dissemination of antibiotic resistance genes is conjugative plasmids. Experimental studies showed that mutations located on the plasmid or the bacterial chromosome can reduce the costs plasmids impose on their hosts, resulting in antibiotic resistance plasmids being maintained even in the absence of antibiotics. While chromosomal mutations are only vertically inherited by the daughter cells, plasmid mutations are also provided to bacteria that acquire the plasmid through conjugation. Here we demonstrate how the mode of inheritance of a compensatory mutation crucially influences the ability of plasmids to spread and persist in a bacterial population.

## INTRODUCTION

Plasmids accelerate bacterial adaptation by transferring ecologically important functions, such as antibiotic resistance, between lineages. Although plasmids can be advantageous in certain environments, their acquisition normally imposes significant fitness costs upon host cells. This is because the expression, repair, and replication of plasmid genes use up raw materials, occupy the cellular machinery, and can disrupt the cellular environment (1, 2). Coculture studies showed that compensatory mutations occurring on plasmids or bacterial chromosomes can reduce these fitness costs of plasmid carriage (3–9), allowing the stabilization of plasmids in the bacterial population (10–12). Because compensatory evolution weakens purifying selection against plasmids, it is likely to increase plasmid persistence even in environments without positive selection for plasmid traits, heightening the risk that plasmid-encoded antibiotic resistances can spread (8, 13, 14).

The mechanisms of amelioration of plasmid costs can be various, including changes in host or plasmid gene expression, conjugation rates, or the loss of plasmid genes (1, 2, 15). Moreover, the extent of the amelioration of the fitness cost varies between compensatory mutations (3–7). Overall, however, compensatory mutations can be considered as reducing the metabolic burden of plasmid carriage, allowing improved bacterial growth but not affecting other processes.

**FIG 1:**
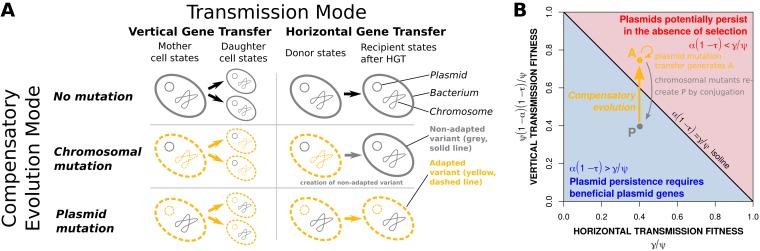
General theoretical concept. (A) The type of compensatory evolution determines the capabilities for the transmission of a compensatory mutation. (B) Plasmid persistence depends on two components characterizing plasmid fitness: (i) the vertical transmission fitness (*y* axis), reflecting the negative effect of the plasmid costs, α, on the host cells’ maximum growth rate, ψ, and the rate of segregational loss, τ (characterizing the inability to inherit the plasmid to both daughter cells by binary fission); and (ii) the horizontal transmission fitness (*x* axis), given by the extent of the conjugation rate, γ. In order to enable comparability, both fitness estimates are normalized to the maximum growth rate, ψ. Plasmids potentially persist in the absence of antibiotic-mediated selection when the combined effects of plasmid costs and segregational loss are lower than the relative extent of conjugation. By compensatory evolution, the initial plasmid costs can be reduced—for instance, by modifying the vertical transmission fitness of a notional plasmid, *P*, to the level of *A*, provided by either a chromosomal mutation or a plasmid mutation. As chromosomal mutants cannot transmit the compensatory mutation horizontally, they generate cells with the original fitness level of *P* when they perform conjugation. For simplicity, we neglect that the amelioration could be coupled to a reduction of the conjugation rate.

We hypothesize that the genomic location of the compensatory mutation, either on the plasmid or the chromosome, will have contrasting effects on plasmid dynamics and persistence: if the compensatory mutation is located on the plasmid, it spreads by both vertical (cell fission) and horizontal (conjugation) transmission. Thus, compensated plasmids acquired by new recipients will impose a reduced cost and be more likely to spread. This likely represents a strong advantage over a chromosomal location (see also the conceptual model presented by Fig. 1A).

In order to comprehensively assess the effects of the genomic location of the compensatory mutation on plasmid dynamics, we developed a mathematical model that simulates the dynamics of plasmid-free bacteria, *F*, nonadapted plasmid bearers, *P*, and adapted plasmid bearers, *A*. The model considers a well-mixed system where (i) bacteria grow with maximal growth rate, ψ, (ii) plasmid bearers suffer according to some plasmids’ costs, α, (iii) compensatory mutations provide an amelioration of these costs by strength β, (iv) bacteria are lost through washout and death according to dilution rate ω and by antibiotic killing rate υ, (v) plasmids are lost during bacterial fission by segregation rate τ, (vi) plasmids are horizontally transferred with conjugation rate γ according to a second-order reaction of plasmid-bearing and plasmid-free cells, and (vii) compensatory mutations are acquired (on either the plasmid or the chromosome) with rate χ (see Table 1 for an overview). (The supplemental material provides more details and a link to a web application of the model and its source code.) Since multiple host strains or species are not considered, we refer to horizontal gene transfer within a nearly clonal cell population, in which intraspecies competition occurs simply between plasmid-free and plasmid-carrying subpopulations. Antibiotic killing does not affect plasmid bearers, because the plasmid confers resistance. A series of simulations were run in order to encompass the full range of plausible plasmid-host properties and environmental conditions, including various levels of antibiotic action.

**TABLE 1:** Model of differential equations describing the dynamics of plasmid-free bacteria and nonadapted and adapted plasmid bearers^*a*^

Process	Bacterial dynamics			Reaction rate
	*F*	*P*	*A*	
Growth	1	0	0	*f*ψ*F*
	0	1	0	*f*ψ(1 − α)*P*
	0	0	1	*f*ψ[1 − α(1 − β)]*A*
Mortality	−1	0	0	(ω + υ)*F*
	0	−1	0	ω*P*
	0	0	−1	ω*A*
Segregation	1	−1	0	*f*τψ(1 − α)*P*
	1	0	−1	*f*τψ[1 − α(1 − β)]*A*
Conjugation	−1	1	0	*f*γ*FP*
	−1	1*^c^*	1*^p^*	*f*γ*FA*
Mutation	0	−1	1	*f*χψ(1 − α)*P*
	0	−1*^p^*	1*^p^*	*f*χγ*FP*

a The model consists of three ordinary differential equations describing the dynamics of plasmid-free bacteria, *F*, nonadapted plasmid bearers, *P*, and adapted plasmid bearers, *A*, respectively. The derivatives of the compartments (*F*, *P*, or *A*) are determined by the reaction rates of the contributing processes, such as growth and mortality. Resource availability: *f *=* *1 – [(*F + P* + *A*)/*k*]. The matrix notation indicates these reaction rates as well as the directions of the particular effects (1, positive; 0, no effect; and −1, negative). Model versions for plasmid mutations and chromosomal mutations differ in two ways. (i) In the first way, conjugation initiated by adapted plasmid bearers, *A*, turns plasmid-free cells, *F*, into bacteria of type *A* (indicated as *p* [superscript]), when the compensatory mutation is located on the plasmid (“plasmid mutation”), or into nonadapted plasmid bearers, *P*, when the mutation is located on the chromosome (“chromosomal mutation,” indicated as *c* [superscript]). Note that *c* is only valid for chromosomal mutation (otherwise 0), and *p* is only valid for plasmid mutation (otherwise 0). (ii) In the second way, compensatory mutations are acquired proportional to replication events, which occur proportional to bacterial growth (given for “chromosomal mutation” and “plasmid mutation”) and conjugation (only given for “plasmid mutation,” *p*). For a parameter description, see Table 2. The equation form of each model version (for “no mutation,” “chromosomal mutation,” and “plasmid mutation”) is presented in equations E1, E2, and E3 in Text S1.

10.1128/mSystems.00186-18.1 TEXT S1 Additional background information and details of the mathematical model, providing the differential equations in formula notation, as well as a description of the single terms for growth, mortality, segregation, conjugation, and adaptation. Download Text S1, PDF file, 0.08 MB. Copyright © 2019 Zwanzig et al. 2019 Zwanzig et al. This content is distributed under the terms of the Creative Commons Attribution 4.0 International license.

## RESULTS

### Cell-inherent requirements for plasmid survival by conjugation.

To disentangle the basic effects of the plasmid- and host-cell-specific characteristics on plasmid survival, we interpret our simulation results in the light of the general theoretical concept presented in Fig. 1B: the competitive disadvantage of plasmid-bearing cells that results from the reduction of the maximal growth rate, ψ, due to the plasmid costs, α, as well as the aligned rate of segregational loss, τ, lead to a decrease of the plasmid-carrying population. The less intense these negative effects are, the higher is a plasmid’s vertical transmission fitness: ψ(1 − α)(1 − τ)/ψ. In contrast, the infection of plasmid-free cells according to conjugation rate, γ, leads to an increase of the plasmid-carrying population. The horizontal transmission fitness controls this process. It increases with the extent of the conjugation rate in relation to the rate at which new plasmid-free cells are generated (γ/ψ). Using these cell-inherent characteristics, we derived a conjugation rate threshold, γ_low_, that approximates the lowest extent of conjugation that can enable plasmid persistence in the absence of selection for plasmid-encoded traits: γ_low_ = αψ(1 − τ).

Plasmids that spread with conjugation rates lower than this threshold and provide no beneficial genes (in our case, in the absence of antibiotics) will not persist. Compensatory evolution can reduce the initial plasmid costs, α, by some amelioration strength, β, to the level of α(1 − β), consequently reducing γ_low_. Nevertheless, plasmids will only persist if cost reduction and conjugation act jointly. Finally, if conjugation rates are higher than γ_low_, a plasmid can survive under certain environmental conditions (see the next section for further details).

### Influence of compensatory evolution on plasmid population dynamics.

To initially test how population dynamics depends on the location of the compensatory mutation, the differential equation model (Table 1) was run using typical parameter values found in literature (Table 2, simulation experiment I).

**TABLE 2:** Model parameters and their settings in simulation experiments^*a*^

Variable	Description	Default value (simulation expt I)	Sampling range (simulation expt II)	Value(s)^*b*^
*k*	Carrying capacity (maximal attainable cell density)	1	1	10^9^ expt→1 for modeling (28)
ψ	Maximal bacterial growth	1	0–2	0.1–1^*c*^ (39), 0.19–1.23^*d*^ (40), 0.74^*e*^ (22)
α	Plasmid costs	0.2	0–1	0.2^*f*^ (41), 0.14–0.19^*g*^ (4), 0.06–0.21^*h*^ (5), 0.21^*i*^ (8), 0.03–0.58 (28), 0.32–0.64^*j*^ (6)
β	Amelioration strength	0.9	0–1	0.1–0.25^*g*^^,^^*l*^ (4), 0.65–1^*i*^^,^^*l*^ (8), 1^*h*^^,^^*j*^^,^^*k*^ (5, 6)
ω	Dilution rate (washout/mortality/predation)	0.1	0–1	0.1 (26, 41)
υ	Antibiotic action (killing rate)	0|10^−1^|10^−2^|10^−3^	10^−4^–1^*p*^	0.1 (8)
τ	Segregation rate	0.001	0.5–10^−6^^*q*^	10^−4^^*i*^ (8), 10^−3^^*e*^ (22)
γ	Conjugation rate	0.02	0–1	0.025^*m*^ (28), 10^−9^^*e*^^,^^*n*^ (22), ≈10^−11^–10^−9^^*n*^ (42)
χ	Mutation rate^*r*^	10^−6^	10^−9^–10^−3^^*p*^	10^−6^ (8), ≈10^−9^–10^−3^ (43), ≈10 − 10^*o*^ (39)

a Simulation experiment I was performed using the parameter defaults in combination with one of the rates for antibiotic action. For simulation experiment II, the sampling range of each parameter defines the parameter space that was used for a random sampling, which generated a compilation of 100,000 parameter sets. Each parameter set was used for simulations with the differential equation model described by the model matrix (Table 1) considering “no mutation” (χ = 0), “chromosomal mutation,” and “plasmid mutation.” To perform simulations in the absence of antibiotics, but under otherwise identical conditions, υ was set to 0, without performing a new sampling.

b Reference numbers are shown in parentheses.

c Estimated for most bacteria in the wild.

d For Escherichia coli, growth on glucose at 17.4 to 40°C.

e Used as simulation standard.

f For plasmid pQBR103.

g For plasmid pBR322.

h For plasmids R1 and RP4.

i For plasmid pNUK73.

j For plasmid R1.

k For coadaptation of host chromosome and plasmid.

l For adaptation of host chromosome.

m Considering relative cell densities.

n Considering absolute cell densities (which can be converted to relative values as used in this study by a multiplication with the respective maximum attainable cell density, *k* [e.g., assuming *k *=* *10^−9^]).

o Laboratory estimates for different species, but mutation rates in the wild are assumed to be higher (39).

p Sampled on a logarithmic scale to prevent an overrepresentation of high values (retransformed by anti-log for simulations).

q Results from uniformly sampling *x* in the range from 1 to 20 and calculating probabilities using function 0.5*^x^*. In this way, τ can also be interpreted as the segregation probability for a certain plasmid copy number *x* (11).

r Referring to a specific compensatory mutation occurring in the first place in the population evolution, but not to neutral, deleterious, or secondary compensatory mutations.

**FIG 2:**
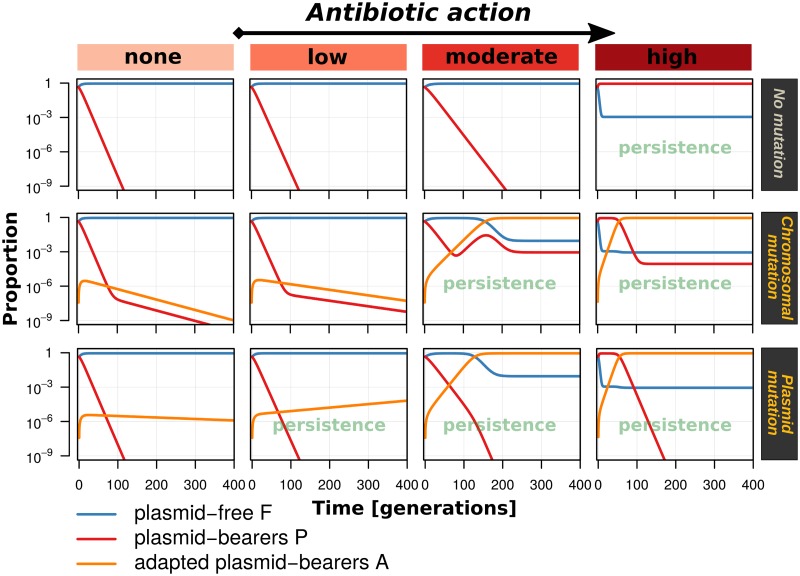
Population dynamics is influenced by the type of compensatory evolution. Each plot refers to a single model run with a set of default parameters (Table 2, simulation experiment I): assuming that plasmid-carriage causes a growth rate disadvantage of 20% (α = 0.2) that can be ameliorated by 90% (β = 0.9), resulting in reduced plasmid costs of only 2% [α(1-β) = 0.02] for adapted plasmid-bearers, considering no mutation, chromosomal mutation, or plasmid mutation, as well as four different levels of antibiotic action, υ (none, 0; low, 10^−3^; moderate, 10^−2^; high, 10^−1^).

Plasmid-bearing bacteria get outcompeted by plasmid-free bacteria unless the level of antibiotic action is sufficiently high (Fig. 2). This effect is generally reduced when compensatory evolution occurs and adapted plasmid bearers with lowered plasmid costs emerge. Although the reduction of plasmid costs is equal in both cases, plasmid mutation enables persistence at lower antibiotic levels than chromosomal mutation.

This is explained by the fact that at low antibiotic concentrations, positive selection is outweighed by the residual cost of plasmid carriage, even following compensatory evolution. Under these conditions, infectious transmission is required to sustain the plasmid in the population. Where compensatory mutations are linked to the bacterial chromosome, conjugation simply increases the proportion of nonadapted plasmid bearers, which suffer the full cost of plasmid carriage and are readily outcompeted. Where compensatory mutations are linked to plasmids, however, newly formed transconjugants suffer a reduced cost of plasmid carriage. Thus, transconjugants expand the proportion of adapted plasmid bearers which support further infectious transmission. The nonadapted plasmid variant is eliminated soon in this case, since it cannot prevail against the more competitive mutant that provides the cost compensation. This feature of a plasmid compensatory mutation represents a fitness advantage (Fig. 1B) that enables plasmid persistence at lower levels of antibiotic-mediated selection.

### Influence of compensatory evolution on the conditions favoring plasmid persistence.

To test whether the observed advantage of plasmid compensatory evolution is stable under a variety of conditions, the deterministic model (Table 1) was repeatedly run using parameter values randomly drawn within reasonable ranges (Table 2, simulation experiment II). Each model run was performed until the proportional changes of plasmid-free, plasmid-bearing, and adapted plasmid-bearing bacteria were less than 10^−9^ (steady state; considering carrying capacity, *k *=* *1).

**FIG 3:**
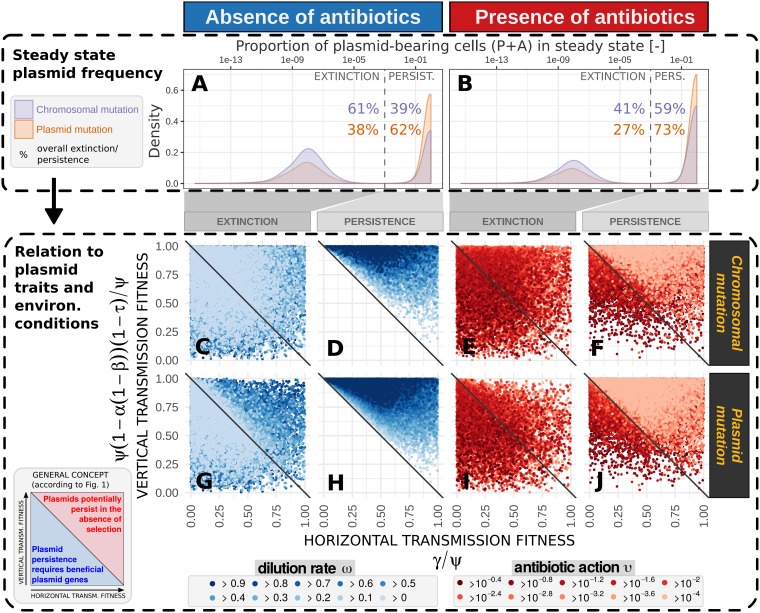
Plasmid compensatory evolution enhances plasmid survival in less favorable environmental settings and fitness contexts. Results refer to the examined global analysis (Table 2, simulation experiment II), where simulations reached a steady state after hundreds to thousands of generations (Fig. S1), characterized by the absence of further substantial changes in the frequencies of plasmid-free bacteria, *F*, plasmid-bearing bacteria, *P*, and adapted plasmid-bearing bacteria, *A*. The prevalence of plasmids in steady state is bimodal (on top). This enabled us to define a threshold (dashed line in plots A and B) to accurately distinguish between long-term plasmid persistence (right from dashed line) and extinction (left from dashed line) and to define the associated overall proportion for chromosomal and plasmid mutation. (Please note that the absolute densities at extinction would all approach −∞, when simulations were not stopped by steady-state computation, i.e., the absence of further substantial changes in the cell proportions.) The conditions that resulted in plasmid extinction and persistence were then further analyzed using the general concept of plasmid fitness introduced in Fig. 1B: Every point in plots C to J refers to the plasmid fitness characteristics (given by the respective parameter values for maximal growth rate, ψ, plasmid costs, α, strength of amelioration, β, segregation rate, τ, and conjugation rate, γ) and the environmental conditions (dilution rate, ω, which refers to washout/natural mortality; and antibiotic action, υ) used in a single model run that resulted either in plasmid extinction or persistence. Since many configurations led to similar results, the degree of overplotting points is high. For this, the order of points is layered from minimal to maximal rates of dilution or antibiotic actions. This feature of the plot reveals the most interesting results regarding plasmid persistence under more unfavorable conditions.

At first, we looked at the resulting frequencies of plasmid-bearing cells in the steady state. The associated pattern is strongly bimodal: plasmids were either prevalent or became (almost) extinct (Fig. 3, top). When the compensatory mutation was located on the plasmid, more plasmids survived compared to a chromosomal mutation.

**FIG 4:**
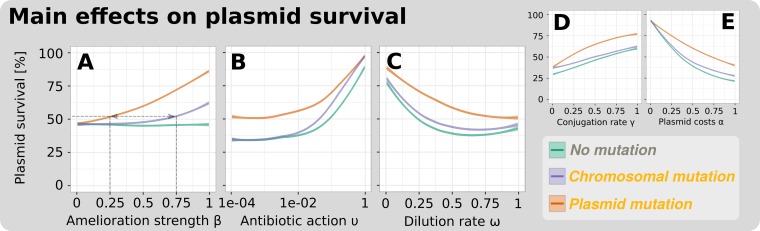
Plasmid compensatory evolution enhances plasmid survival across the full range of tested plasmid properties and environmental conditions. Results refer to the global sensitivity analysis considering the presence of antibiotics (Table 2, simulation experiment II). The single panels depict the main effects of each parameter across its range for each type of compensatory evolution. The higher the difference between the lowest and highest plasmid survival percentages, the stronger is the main effect of this parameter. Antibiotic action (B) and plasmid costs (E) have the strongest main effect. Nonlinear responses indicate that certain parameter ranges are more sensitive than others (when the response is steeper). The slight reversal to increased plasmid survival percentages at high dilution rates results from the extreme conditions for antibiotic-sensitive, plasmid-free cells, when the combined effects of dilution and antibiotic action approach 1. Main effects of the remaining parameters are depicted in Fig. S2.

10.1128/mSystems.00186-18.3 FIG S1 Characterization of the steady state of all simulations run in simulation experiment II (Table 2) by persistence time and proportion of plasmid-bearing cells. Each point reflects the conditions when a numerical steady state is reached for a given combination of plasmid-host properties and environmental conditions. Columns indicate the results for simulations considering the absence (left) or presence (right) of antibiotics. Rows distinguish the results for the types of compensatory evolution (“no mutation,” “chromosomal mutation,” and “plasmid mutation”), which differ only slightly on this scale. It can also be seen that the prevalence of plasmids in steady state is bimodal, which enabled us to define a threshold (dashed line) to accurately distinguish between long-term plasmid persistence (right from dashed line) and extinction (left from dashed line). (A similar representation is shown in Fig. 4.) Download FIG S1, TIF file, 2.9 MB. Copyright © 2019 Zwanzig et al. 2019 Zwanzig et al. This content is distributed under the terms of the Creative Commons Attribution 4.0 International license.

We further examined how the result of plasmid extinction or persistence is related to plasmid traits and environmental conditions. In the absence of antibiotics, plasmids could only survive when the threshold γ_low_ was met. In this case, the competitive disadvantage of the remaining plasmid costs, α(1 − β), and the proportional rate of plasmid loss, τ, can be outweighed by the relative extent of the conjugation rate, γ: thus, {ψ[1 − α(1 − β)(1 − τ)]/ψ} < γ/ψ (Fig. 3D and H). Plasmids could not survive by exerting conjugation rates below the threshold γ_low_, which refers to any conditions below the diagonal line in the Fig. 3D and H. This result is in line with our preliminary theoretical considerations (Fig. 1B or Fig. 3, bottom left). As further expected, plasmids also got lost when the threshold γ_low_ was met (conditions above the diagonal line in Fig. 3C and G), since this threshold represents an exclusion criterion rather than a stability criterion. With an increased dilution, more bacteria are lost—mimicking washout, natural mortality, predation, or a combination of these processes. This results in decreased bacterial densities that lower the real efficiency of the conjugation rate. The potential of plasmid persistence (even above γ_low_) therefore increases with lower dilution rates and higher vertical transmission (low plasmid costs and segregation rates), respectively.

In the presence of antibiotics, plasmids were able to persist even below the threshold γ_low_ (conditions below the diagonal line in Fig. 3F and J), at least when antibiotic killing rates, υ, were high, representing a strong selection on the resistant plasmid bearers. Nevertheless, some plasmids got lost even if they met the requirements given by the threshold γ_low_ (i.e., by high conjugation rates and/or low plasmid costs) and even if they were supported by high antibiotic action (conditions below the diagonal line in Fig. 3E and I [please consider that the orders of point layers are different between panels F and J and E and I]). This traces back to the effect of dilution, which can therefore also impact plasmid survival in the presence of antibiotics. It suggests that, e.g., higher levels of predation are likely to drive antibiotic resistance genes out, at least in the well-mixed phase.

Most interestingly, plasmid compensatory evolution enabled the persistence of plasmids under less favorable conditions: i.e., inferring higher plasmid costs, exerting lower conjugation rates, and/or facing higher dilution rates or weaker antibiotic-mediated selection. This advantage of a plasmid compensatory mutation over a chromosomal compensatory mutation especially manifests at high conjugation rates, since chromosomal mutants cannot transfer the cost compensation to horizontally infected cells (Fig. 1), whereas plasmid bearers that carry and transfer the cost compensation via the plasmid also to any infected cell directly benefit from conjugation.

To test if the effect of compensatory evolution on plasmid persistence is particularly sensitive to a single parameter of our model, we examined the probability of plasmid persistence for any parameter across the ranges that were used in our simulations. Across the majority of the parameter, space compensatory mutations increased the probability of plasmid persistence (see Fig. 4 for most important parameters and Fig. S2 in the supplemental material for the remaining parameters). This effect was much greater when mutations were linked to the plasmid rather than the chromosome. This demonstrates that the advantage of plasmid mutations is robust within the broad range of tested parameter values. Although the mutation rate does not affect the steady-state results of our model (Fig. S2), it should be noted that it accelerates the increase of adapted plasmid bearers in the short term (see Fig. S3 in the supplemental material) and might represent an advantage of chromosomal mutations (see Discussion).

10.1128/mSystems.00186-18.4 FIG S2 Nonlinear main effects of remaining model parameters not shown in Fig. 4 in the main text. Results refer to the global analysis considering the presence of antibiotics (Table 2, simulation experiment II). Here, it is consecutively examined how the variation of a single parameter within its predefined range affects the overall result of all tested conditions. Download FIG S2, TIF file, 0.7 MB. Copyright © 2019 Zwanzig et al. 2019 Zwanzig et al. This content is distributed under the terms of the Creative Commons Attribution 4.0 International license.

10.1128/mSystems.00186-18.5 FIG S3 Increasing mutation rates accelerate plasmid prevalence. Rows refer to the types of compensatory evolution (“chromosomal mutation” and “plasmid mutation”) and columns to increasing mutation rates, χ, namely 10^−9^, 10^−6^, and 10^−3^. All other parameters are as the default (Table 2), with antibiotic action as υ = 10^−2^. Overall, an increase of χ by as much as 10^6^ results only in a 3-fold increase (here around 160 generations) of the time until plasmids reach their most prevalent state. Download FIG S3, TIF file, 0.7 MB. Copyright © 2019 Zwanzig et al. 2019 Zwanzig et al. This content is distributed under the terms of the Creative Commons Attribution 4.0 International license.

Overall, plasmid compensatory evolution increased the proportion of conditions enabling plasmid persistence far more by increased amelioration strength (Fig. 4A). Even by providing only 25% reduction of the plasmid costs, plasmid mutation allowed persistence for the same proportion of conditions as mediated by chromosomal mutations, with a cost compensation of 75%. The advantage of plasmid mutations that is given by the ability to transfer the cost compensation with the plasmid to both daughter and infected cells also manifests by higher survival rates at very low antibiotic action.

## DISCUSSION

In this article, we examined the role of two different types of compensatory evolution, namely chromosomal mutations versus plasmid mutations, for the long-term persistence of plasmids. We demonstrated that

1. Compensatory evolution can have a significant effect, allowing plasmid-encoded antibiotic resistances to persist for a much longer time, even in the absence of antibiotics.
2. The genomic location (chromosome or plasmid) can play a pivotal role for the success of a compensatory mutation. Since chromosomal mutations cannot be transmitted to infected bacteria, the benefits that such plasmid-bearing bacteria could gain from conjugation are reduced.
3. Plasmid mutations facilitate plasmid persistence even when the direct amelioration effects are far less effective than those provided by chromosomal mutations.

If the plasmid adapts, it is also very likely that the nonadapted plasmid variant will be eliminated soon, as it cannot prevail against the more competitive mutant that provides the cost compensation. Of course, this may not occur, if a more specific mechanism of compensatory evolution is considered, e.g., the reduction of the conjugation rate or the loss of functional traits (1), which would reshape the fitness differences between nonadapted and adapted plasmid bearers. A deletion of the type 4 secretion system (T4SS) or a part of it can decrease the costs associated with plasmid carriage (16), but any increase of vertical transmission to the detriment of horizontal transmission implies a shift in a plasmid’s survival strategy. This can sustain a resistance plasmid in its current host, even in the absence of selection (5), but likely limits its dissemination potential to new hosts. Understanding this trade-off in interplay with the genomic location of the compensatory mutation represents a further challenge, which can also be addressed with mathematical methods analogous to those used in this study.

It should be noted that compensatory mutations are discrete events that arise on the single-cell or plasmid level. In small bacterial populations, the resulting stochasticity needs to be considered (17). Although it is hard to predict which mutations might be available to ameliorate the plasmid costs, chromosomal mutations might occur much more frequently than plasmid mutations, considering their different amounts of genes. This timing can be important to stabilize the plasmid before it is lost from the population (17) and might explain why chromosomal mutations are so common in experimental evolution.

Our results indicate that a single nontransmissible plasmid cannot persist in the absence of selection of plasmid-borne traits, but this might not hold considering a more diverse plasmid community, since conjugative plasmids can promote the survival of co-occurring, less costly nontransmissible plasmids of the same incompatibility group (18). It is also important to note that the availability of alternative hosts, which is not considered in our model, can have evolutionary consequences on plasmid population dynamics. This is because fitness effects of the same plasmid can be really variable in different hosts (19), although some plasmid compensatory mutations have been shown to increase plasmid persistence even in other plasmid-host pairs (20). Another effect of a multispecies (or nonclonal) host environment is that interspecific plasmid transfer can allow plasmids to survive in host species which cannot sustain the plasmid in monoculture (21). Furthermore, we neglected that bacteria experience spatiotemporal fluctuating environmental conditions during their lifetime, which could help to sustain a resistance plasmid by rare antibiotic exposure (8).

Our model considers average conditions in notional habitats. Such simple mass action models have been successfully applied to various research questions related to plasmid biology (22–28) as they allow a fast computation of highly comprehensive simulation experiments, which can be used to draw general conclusions (29). Our results are in line with model-supported empirical studies highlighting the importance of conjugation-assisted persistence for costly plasmids (28) and provide further insights into the role of compensatory evolution for the empirically found persistence of antibiotic resistances at concentrations of antibiotics far below the MIC of the susceptible strain (30, 31).

We believe that a more thorough exploration of this issue, especially regarding to the mechanisms that underlie the costs of plasmid carriage and the conditions in natural microbial communities, will be an important further step toward an improved understanding of the population dynamics and evolutionary biology of plasmids. This will also help to develop strategies against the dissemination of antibiotic resistance genes.

## MATERIALS AND METHODS

### Mathematical model.

Our model consists of a system of three ordinary differential equations. It describes the dynamics of the following compartments: plasmid-free bacteria, *F*; plasmid-bearing bacteria, *P*; and adapted plasmid-bearing bacteria, *A.* The latter originates from the evolution of the plasmid and its host and is provided either by a mutation of the chromosome or a mutation of the plasmid (in the meaning of a compensatory mutation occurring in the first place in the population evolution; neutral, deleterious, or secondary compensatory mutations are not considered). To compare both types of compensatory evolution, we developed two different model versions. They are described as a model matrix (Table 1). All parameters and their settings in the simulation experiments are given in Table 2.

Please note that this study considered relative cell densities instead of absolute ones. This enables a direct comparison of the extent of plasmid costs and conjugation. If absolute cell densities would be used, the conjugation rate has to be scaled in relation to the carrying capacity, *k*. Assuming, for example, γ = 0.2 and *k *=* *10^9^ cells/ml, an adjusted conjugation rate, γ_adjusted_, of γ/*k *=* *2e−11 would be obtained. This means the model could also be run with typical conjugation rates to those generated by the endpoint method (32). Model performance would remain the same as long as robust estimates for *k* are available.

Our model does not consider that the viability of resistant cells (plasmid bearers *P* or *A*) can be reduced by the antibiotic (33). We assume this to be a valid simplification, since the bactericidal antibiotic killing rate was for instance reported to be 55 times higher for sensitive bacteria than for resistant bacteria (8). As we consider antibiotic action to range only from no inhibition (υ = 0) to full inhibition of the plasmid-free bacteria (υ = 1), we never achieve those levels that cause, with respect to dose-response experiments (8), a significant inhibition of resistant bacteria. Our model predictions are therefore valid in this range.

The supplemental material (Text S1) provides further details of the model and the link to a web application that enables the reader to explore the model behavior with default or self-defined parameter estimates. (Please, consider the discussion on relative and absolute conjugation rates above.)

### Simulation experiments.

Two different types of simulation experiments were carried out. While simulation experiment I focused on the general dynamics of the model system with a reference parameter set, simulation experiment II scanned the whole parameter space in order to reveal which conditions favor the long-term persistence of plasmids.

Both experiments have in common that the initial frequency of plasmid-free bacteria, *F*, and nonadapted plasmid bearers, *P*, was defined as half of the proportion of bacteria that are approached in steady state, which was approximated by *k*(1 − ω). Since the latter depends on the particular parameter settings, the initial frequency could differ in absolute values, but the *F*/*P*_initial_ ratio remained the same. Adapted plasmid bearers, *A*, were not present in the initial state. Please note that model results are insensitive to the particular initial conditions (see Fig. S4 in the supplemental material for validation).

10.1128/mSystems.00186-18.6 FIG S4 Mathematical phase planes for the population dynamics considering “no mutation,” “chromosomal mutation,” and “plasmid mutation.” Curves show the trajectories from random initial relative frequencies of plasmid-free cells, *F*, and nonadapted plasmid bearers, *P*. Red dots mark the stable fixed points to which the trajectories are attracted, which demonstrates that the steady-state results are independent from the initial conditions. Parameters are as the default (Table 2), and antibiotic action is set to a moderate level (υ = 10^−2^). All model versions show the same behavior and turn to the same attraction point in the beginning, but the evolution of adapted plasmid bearers, *A*, given by a compensatory mutation located on the chromosome or the plasmid, causes the movement to a reverse attraction point and enables the persistence of the plasmid. Download FIG S4, TIF file, 0.9 MB. Copyright © 2019 Zwanzig et al. 2019 Zwanzig et al. This content is distributed under the terms of the Creative Commons Attribution 4.0 International license.

**(i) Simulation experiment I: general dynamics of the model system.** Simulation runs were carried out with default parameter values (Table 1), considering the absence of antibiotics as well as their presence at low, moderate, and high levels. The time horizon of one simulation run corresponded to a time course of 4,000 h (here equal to approximately 400 generations).

**(ii) Simulation experiment II: conditions favoring the long-term persistence of plasmids and associated antibiotic resistances.** Simulation experiments were carried out within the parameter space of reasonable maxima and minima (Table 2, third column) mimicking a broad spectrum of possible plasmid-host properties and environmental conditions. Each parameter was randomly sampled generating 10,000 parameter sets by an adjusted Latin hypercube approach (34).

The objective of any of these simulations was to examine if the proportion of plasmid bearers (*P + A*) was still higher than 10^−3^ when the system reached a steady state. A steady state was assumed when the proportional change in the population composition was less than 10^−9^ h^−1^. This corresponds to a change of the bacterial composition of less than 1 cell per hour for an absolute carrying capacity of 10^9^ cells.

Long-term persistence of plasmids was assumed when the proportion of plasmid bearers (*P + A*) was still higher than 10^−3^ after reaching the steady state. The threshold of 10^−3^ was arbitrarily chosen but served well for the distinction between settings promoting persistence and leading to extinction since the vast majority of simulation experiments leading to the latter ended up with plasmid bearer proportions of less than 10^−6^ (see Fig. S1 for a validation of the system’s behavior).

### Model implementation.

The model was implemented in the software environment R version 3.4.2 (35) and executed using aligned packages deSolve (36) and rootSolve (37) for solving and analyzing the steady state of ordinary differential equations. Sampling in simulation experiment II was performed with function randomLHS from R package lhs (34). Graphics were generated using R package ggplot2 (38) and core functions of R. The source code is available in the supplemental material (Text S2).

10.1128/mSystems.00186-18.2 TEXT S2 The computer code of the mathematical model, representing a platform to perform simulations with our model called PlasSim (for plasmid population dynamics simulator), equal to the interactive web app at https://martin20.shinyapps.io/plassim/, which can also be used to download the results of simulations that were run with self-defined parameters. Download Text S2, TXT file, 0.02 MB. Copyright © 2019 Zwanzig et al. 2019 Zwanzig et al. This content is distributed under the terms of the Creative Commons Attribution 4.0 International license.
