# Supplementary material for: Mobile Compensatory Mutations Promote Plasmid Survival
Source: mSystems. 2019 Jan 15;4(1):e00186-18. doi: 10.1128/mSystems.00186-18 (PMC6446977; doi:10.1128/mSystems.00186-18)
Supplement: TEXT S1 [file sys001192313s1.pdf]

Supplemental Material

Mobile compensatory mutations promote plasmid survival

Martin Zwanzig (orcid.org/0000-0003-1866-6743) [a,1], Ellie Harrison [b], Michael A. Brockhurst [b], James P. J. Hall [c], Thomas U. Berendonk [d], and Uta Berger [a]

[a] Department of Forest Sciences, Institute of Forest Growth and Forest Computer Science, Technische Universität Dresden, Tharandt, 01735, Germany; [b] Department of Animal and Plant Sciences, University of Sheffield, Sheffield S10 2TN, UK; [c] Department of Evolution, Ecology and Behaviour, Institute of Integrative Biology, University of Liverpool, Liverpool L69 7ZB; [d] Department of Hydro Sciences, Institute of Hydrobiology, Technische Universität Dresden, Dresden, 01217, Germany

[1] To whom correspondence should be addressed. E-mail: martin.zwanzig@tu-dresden.de

Supplemental text

This text provides a more detailed description of the model system. Please note that we deployed our model using the *R*-package shiny (1) and the hosting service 'shinyapps.io', which allows the user to parameterize, execute and explore our model and the following simulation results by an interactive web app (https://martin20.shinyapps.io/plassim/). Since we want to be fully transparent, we also provide the full code, which enables you to check the model and to run it on your local machine (source code S1).

Additional Background information

The conditions enabling plasmid persistence have been addressed in a series of simulation studies reflecting theoretical as well as empirical considerations (2-10). Although there was a long debate about horizontal transfer rates, recent studies demonstrate that common conjugative plasmids are indeed transferred at sufficiently high rates to stabilize the plasmid in the population, even if plasmid costs are high and the environment does not select for plasmid-encoded traits such as mercury resistances (8) or antibiotic resistances (10).

In this study we consider the whole continuum of plasmid traits and environmental conditions, including high or low infectivity, plasmid costs and selection regimes. This allows us to obtain general insights and to explore the 'plasmid fitness space' (Fig. 1 B), which we refer to both components of a plasmid’s fitness (11): (i) its vertical transmission fitness, given by the ability to spread within the same cellular lineage and (ii) its horizontal transmission fitness, given by the ability to infect new hosts through conjugation.

Common fitness estimates resulting from empirical measurements often either reflect a comparison of single growth rates or they compare initial and final population sizes of two competing strains or populations (12). In this study we like to adapt this concept and define fitness as a relative estimate by comparing both (i) the growth rates of plasmid-bearers and (ii) their conjugation rates to the growth rates of plasmid-free cells. This estimates how much the single components affect the proliferation of the plasmid-bearers in the next generation. Whereas the relative contribution of conjugation to plasmid fitness often remains inconclusive from empirical studies (12, 13), our mathematical methods enable an exact definition.

Details of the mathematical model

Our model represents a system of ordinary differential equations describing the dynamics of plasmid-free bacteria F, non-adapted plasmid-bearers P and adapted plasmid-bearers A according to growth, dilution (by washout/mortality/predation), segregation, conjugation and compensatory evolution. Equations are presented in a succinct matrix in Table 1 and parameters are described in Table 2 in the main text. The differential equations considering each type of compensatory evolution (‘no mutation’, ‘chromosomal mutation’ and ‘plasmid mutation’) can also be written as follows:

Considering no compensatory evolution (‘no mutation’), only plasmid-free cells F and (non-adapted) plasmid-bearers P compete (the mutation rate χ is assumed to be 0 and no adapted plasmid-bearers A are generated):

$$\frac{dF}{dt} = f\left(\psi F + \tau\psi\left(1-\alpha\right)P - \gamma FP\right) - \left(\omega+\upsilon\right)F$$
$$\frac{dP}{dt} = f\left(\psi\left(1-\alpha\right)P - \tau\psi\left(1-\alpha\right)P + \gamma FP\right) - \omega P$$
(Equ. E1)

with resource availability $f = 1 - \frac{F+P}{k}$, carrying capacity k, maximal growth rate ψ, plasmid costs α, segregation rate τ and conjugation rate γ.

Considering compensatory mutations located on the chromosome (‘chromosomal mutation’), adapted plasmid-bearers emerge proportional to fission with process rate $\chi\psi(1\text{-}\alpha)P$ and grow with plasmid costs reduced by amelioration strength β, whereas the compartment of non-adapted plasmid-bearers P benefits from the infection of plasmid-free cells by A (γFA), since A cannot transmit the chromosomal mutation by conjugation:

$$\frac{dF}{dt} = f\left(\psi F + \tau\psi\left(1-\alpha\right)P + \tau\psi\left(1-\alpha\left(1-\beta\right)\right)A - \gamma FP - \gamma FA\right) - \left(\omega+\upsilon\right)F$$
$$\frac{dP}{dt} = f\left(\psi\left(1-\alpha\right)P - \tau\psi\left(1-\alpha\right)P + \gamma FP + \gamma FA - \chi\psi\left(1-\alpha\right)P\right) - \omega P$$
$$\frac{dA}{dt} = f\left(\psi\left(1-\alpha\left(1-\beta\right)\right)A - \tau\psi\left(1-\alpha\left(1-\beta\right)\right)A + \chi\psi\left(1-\alpha\right)P\right) - \omega A$$
(Equ. E2)

with resource availability $f = 1 - \frac{F+P+A}{k}$, and, in addition to those listed for Equ. E1, amelioration strength β (of compensatory mutation) and mutation rate χ.

Considering compensatory mutation located on the plasmid (‘plasmid mutation’), the compartment of adapted plasmid-bearers A benefits from the rate γFA (transmission of the compensatory mutation with the plasmid to the infected plasmid-free cells F) and $\chi\gamma FP$ (mutation proportional to plasmid replication after conjugation):

$$\frac{dF}{dt} = f\left(\psi F + \tau\psi\left(1-\alpha\right)P + \tau\psi\left(1-\alpha\left(1-\beta\right)\right)A - \gamma FP - \gamma FA\right) - \left(\omega+\upsilon\right)F$$
$$\frac{dP}{dt} = f\left(\psi\left(1-\alpha\right)P - \tau\psi\left(1-\alpha\right)P + \gamma FP - \chi\psi\left(1-\alpha\right)P - \chi\gamma FP\right) - \omega P$$
$$\frac{dA}{dt} = f\left(\psi\left(1-\alpha\left(1-\beta\right)\right)A - \tau\psi\left(1-\alpha\left(1-\beta\right)\right)A + \gamma FA + \chi\psi\left(1-\alpha\right)P + \chi\gamma FP\right) - \omega A$$
(Equ. E3)

The features of the biological system we like to capture with our mathematical model are those of a well-mixed aquatic system. In laboratory experiments this is often mimicked by the use of chemostats, which enable a stabilization and homogenization of certain environmental conditions. In such systems bacteria can grow in a physiological steady state, with a specific growth rate that depends on a species- and substrate-dependent maximal growth rate, here denoted as ψ. Furthermore, washout and death of bacteria determines the loss of bacteria from the system domain, here denoted as the dilution rate ω. If $\psi < \omega$ all bacteria will be washed out, because the bacterial population cannot sustain itself. Otherwise, if $\psi > \omega$ the bacterial population will reach a constant specific growth rate at steady state that is equivalent to the dilution rate ω, because then, when the system approaches the stationary phase, growth is limited by the proportion of bacteria that become washed out or die. In our model, this competition for resources is considered explicitly. Any resource-dependent processes are assumed to be limited by a carrying capacity k, modeled according to the standard logistic law, given by the factor f in the reaction rates in Table 1 and equations E1, E2 and E3. This approach has been previously applied for growth in a plasmid population model (6), but we also account for a resource-dependency of conjugation, which has been highlighted in many studies (3, 14-21), and the influence of resource-dependent growth and conjugation on segregation and compensatory evolution. It is important to account for a resource dependency of conjugation, since we would otherwise assume that bacteria are infected with the same rate, even if bacterial growth approaches zero, when resources are exhausted.

For convenience, we refer to relative instead of absolute densities in our model. This means bacterial densities of F, P and A range between 0 and 1 and the carrying capacity k is always 1, i.e. equals the maximal relative cell density.

Growth

Plasmid-free bacteria F grow with respect to a maximal growth rate ψ, whereas plasmid-bearers P suffer according to some plasmid costs α, that can be compensated to a certain extent for adapted plasmid-bearers A by amelioration strength β.

Mortality

All three compartments *F*, *P* and *A* are influenced by a dilution rate ω, which represents an unspecific homogeneous reduction of the entire population that can be referred to natural mortality, bacterial washout, predation pressure or the combination of these effects. It is assumed that only plasmid-free bacteria *F* are sensitive to the action of a bactericidal antibiotic, given by the antibiotic killing rate υ. This implies that plasmids remain the sole source of antibiotic resistance genes or that only plasmid carriage and the associated multiplicity of resistance genes enables a sufficient degree of resistance.

Segregation

The probability to produce a plasmid-free daughter cell during bacterial fission is considered to be a random event, represented by the probability τ. In nature this probability can be linked to the plasmid copy number, in the following denoted as *c*. Assuming that segregation is totally random, the probability for segregation could also be calculated by 0.5^{c-1} (11). Although this is an oversimplification, as certain mechanisms can prevent plasmid loss, it enables us to evaluate the effect of certain degrees of plasmid instability. For the sake of simplicity, and because the test of an extended model version revealed that the associated effect is marginally, it is further assumed that all plasmid-bearers that lose their plasmid turn into plasmid-free cells, even those bacteria that acquired a chromosomal mutation.

Conjugation

Plasmid transfer in mixed cultures can be described by simple mass-action models (2, 4, 14, 22, 23). Whereas growth, mortality and segregation are first-order reactions, conjugation follows the law of mass action as a second-order reaction of plasmid-bearing and plasmid-free cells. This assumes that (i) mating occurs at random with a frequency proportional to the joint frequency of bacteria, (ii) the ratio of plasmid-free to plasmid-bearing bacteria is not significant, (iii) the time since last receipt or transfer has no effect on transfer rates and (iv) all bacteria or plasmids of the same type have

identical properties. Although these are simplifying model assumptions, they have already been demonstrated to be applicable to both broth and chemostat experiments for varying plasmid types (14). Similarly, the conjugation rate in our model is defined by a single parameter γ (Table 1).

The results presented in the main text were generated using relative cell densities, which also assumed that the carrying capacity *k* equals one. If instead absolute values for cell densities and the carrying capacity are given, for instance in cells / ml, the conjugation rate has to be scaled relative to the carrying capacity *k* in order to generate comparable results:

$\gamma_{abs} = \gamma / k$

The resulting estimates for γ_{abs} reflect typical values as those generated by the end-point-method (22). For example utilizing our default parameter estimates (Table 2) and assuming $k = 10^9$ we obtain a conjugation rate $\gamma = 2e^{-11}$.

Nevertheless, in this study we considered relative cell densities to account for the relative extent of both plasmid costs and conjugation. This enabled us to directly compare the conjugation rate γ with the plasmid costs α in relation to the hosts maximal growth rate ψ.

Adaptation

In the initial state, only plasmid-free bacteria *F* and non-adapted plasmid-bearers *P* are present. Compensatory evolution by chromosomal or plasmid mutations leads to the emergence of adapted plasmid-bearers *A*. It is assumed that beneficial mutations are acquired with a rate χ in the course of replication events. Whereas the bacterial chromosome replicates during fission, plasmid replication takes place according to fission and conjugation.

In this study, the conjugation rate γ does not change as an effect of compensatory evolution. This means γ is fixed at the same level for both non-adapted plasmid-bearers *P* and adapted plasmid-bearers *A* within a single simulation run. Changing this feature might convey interesting results in further investigations.

Supplemental Material References

1. Chang W, Cheng J, Allaire J, Xie Y, McPherson J (2017) shiny: Web Application Framework for R. R package version 1.0.5.

2. Stewart FM, Levin BR (1977) The population biology of bacterial plasmids: A priori conditions for the existence of conjugationally transmitted factors. Genetics 87(2):209–228.

3. Simonsen L (1991) The existence conditions for bacterial plasmids: Theory and reality. Microbial Ecology 22(1):187–205.

4. Bergstrom CT, Lipsitch M, Levin BR (2000) Natural selection, infectious transfer and the existence conditions for bacterial plasmids. Genetics 155(4):1505–1519.

5. Willms AR, Roughan PD, Heinemann JA (2006) Static recipient cells as reservoirs of antibiotic resistance during antibiotic therapy. Theoretical Population Biology 70(4):436–451.

6. Lili LN, Britton NF, Feil EJ (2007) The persistence of parasitic plasmids. Genetics 177(1):399–405.

7. Harrison E, et al.Dytham, C, Hall, JPJ, Guymer, D, Spiers, AJ, Paterson, S, Brockhurst, MA (2016) Rapid compensatory evolution promotes the survival of conjugative plasmids. Mobile Genetic Elements 6(3):e1179074.

8. Hall JPJ, Wood AJ, Harrison E, Brockhurst MA (2016) Source–sink plasmid transfer dynamics maintain gene mobility in soil bacterial communities. Proceedings of the National Academy of Sciences 113(29):8260–8265.

9. Hall JPJ, Brockhurst MA, Dytham C, Harrison E (2017) The evolution of plasmid stability: Are infectious transmission and compensatory evolution competing evolutionary trajectories? Plasmid 91:90–95.

10. Lopatkin AJ, et al. Meredith, HR, Srimani, JK, Pfeiffer, C, Durrett, R, You, L (2017) Persistence and reversal of plasmid-mediated antibiotic resistance. Nature Communications 8(1):1689.

11. Summers DK (1996) The Biology of Plasmids. (Blackwell Science Ltd).

12. Wiser MJ, Lenski RE (2015) A comparison of methods to measure fitness in escherichia coli. PLoS ONE 10(5):e0126210.

13. San Millan A and MacLean RC (2017) Fitness costs of plasmids: a limit to plasmid transmission. Microbiol Spectrum 5(5):MTBP-0016-2017.

14. Levin BR, Stewart FM, Rice VA (1979) The kinetics of conjugative plasmid transmission: Fit of a simple mass action model. Plasmid 2(2):247–260.

15. Freter R, Freter RR, Brickner H (1983) Experimental and mathematical models of escherichia coli plasmid transfer in vitro and in vivo. Infection and Immunity 39(1):60–84.

16. MacDonald JA, Smets BF, Rittmann BE (1992) The effects of energy availability on the conjugative-transfer kinetics of plasmid rp4. Water Research 26(4):461–468.

17. Fox RE, Zhong X, Krone SM, Top EM (2008) Spatial structure and nutrients promote invasion of IncP-1 plasmids in bacterial populations. ISME J 2(10):1024–1039.

18. Philipsen KR, Christiansen LE, Hasman H, Madsen H (2010) Modelling conjugation with stochastic differential equations. Journal of Theoretical Biology 263(1):134–142.

19. Haagensen JAJ, Hansen SK, Johansen T, Molin S (2002) In situ detection of horizontal transfer of mobile genetic elements. FEMS Microbiology Ecology 42(2):261–268.

20. Seoane J, Yankelevich, T, Dechesne, A, Merkey, B, Sternberg, C, Smets, BF (2011) An individual-based approach to explain plasmid invasion in bacterial populations. FEMS Microbiology Ecology 75(1):17–27.

21. Merkey BV, Lardon LA, Seoane JM, Kreft JU, Smets BF (2011) Growth dependence of conjugation explains limited plasmid invasion in biofilms: an individual-based modelling study. Environmental Microbiology 13(9):2435–2452.

22. Simonsen L, Gordon DM, Stewart FM, Levin BR (1990) Estimating the rate of plasmid transfer: an end-point method. Journal of General Microbiology 136(11):2319–2325.

23. Zhong X, Droesch J, Fox R, Top EM, Krone SM (2012) On the meaning and estimation of plasmid transfer rates for surface-associated and well-mixed bacterial populations. Journal of Theoretical Biology 294(0):144–152.
