# Supplementary figures and images for: Mobile Compensatory Mutations Promote Plasmid Survival
Source: mSystems. 2019 Jan 15;4(1):e00186-18. doi: 10.1128/mSystems.00186-18 (PMC6446977; doi:10.1128/mSystems.00186-18)

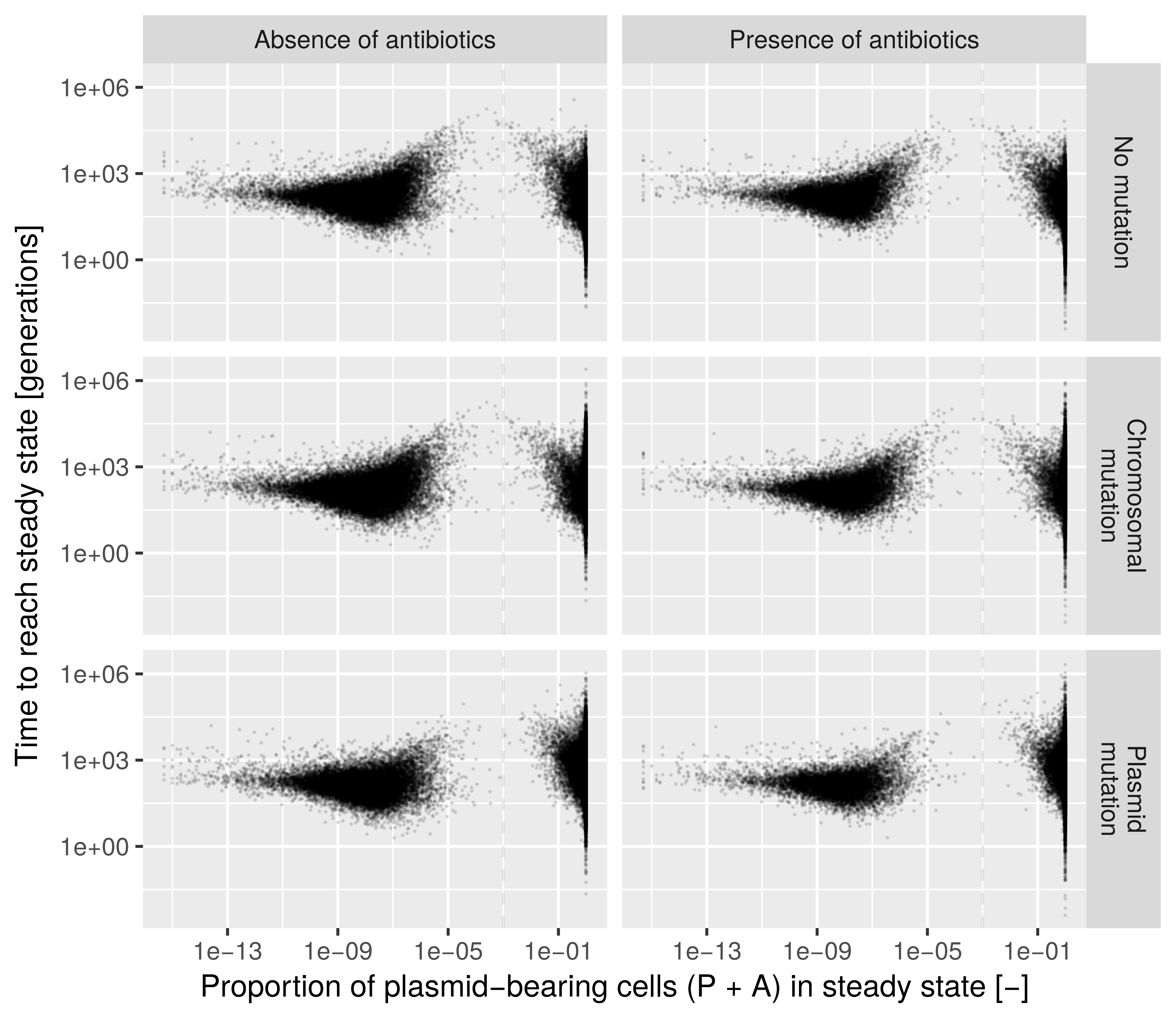

Supplement: FIG S1 [file sys001192313sf1.tif]

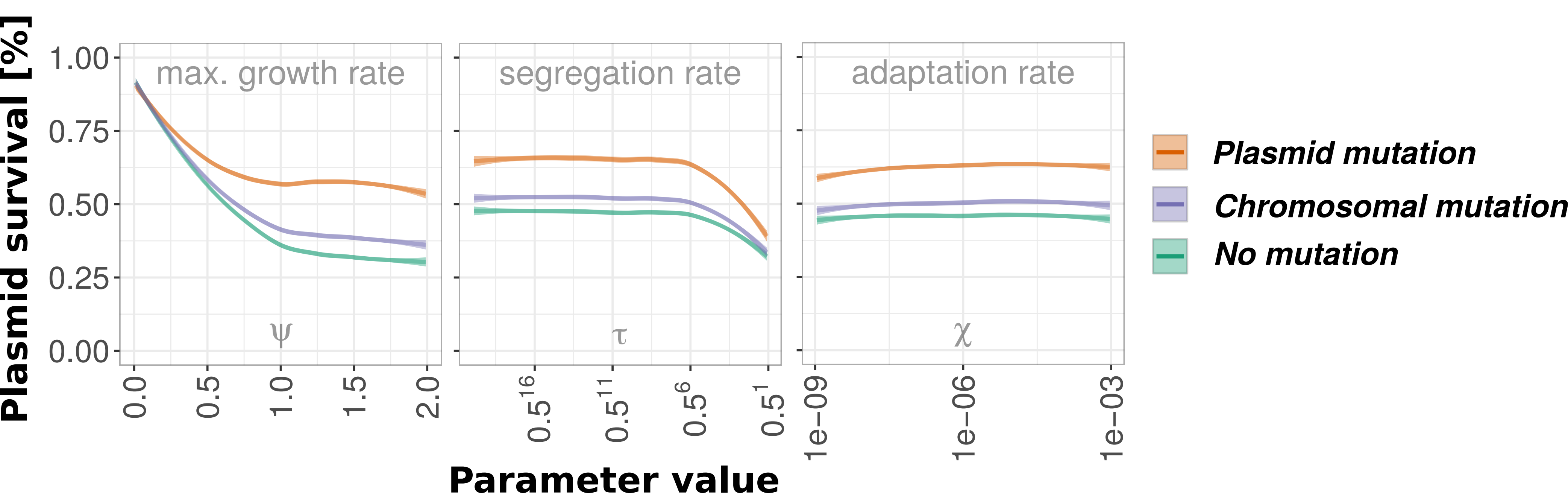

Supplement: FIG S2 [file sys001192313sf2.tif]

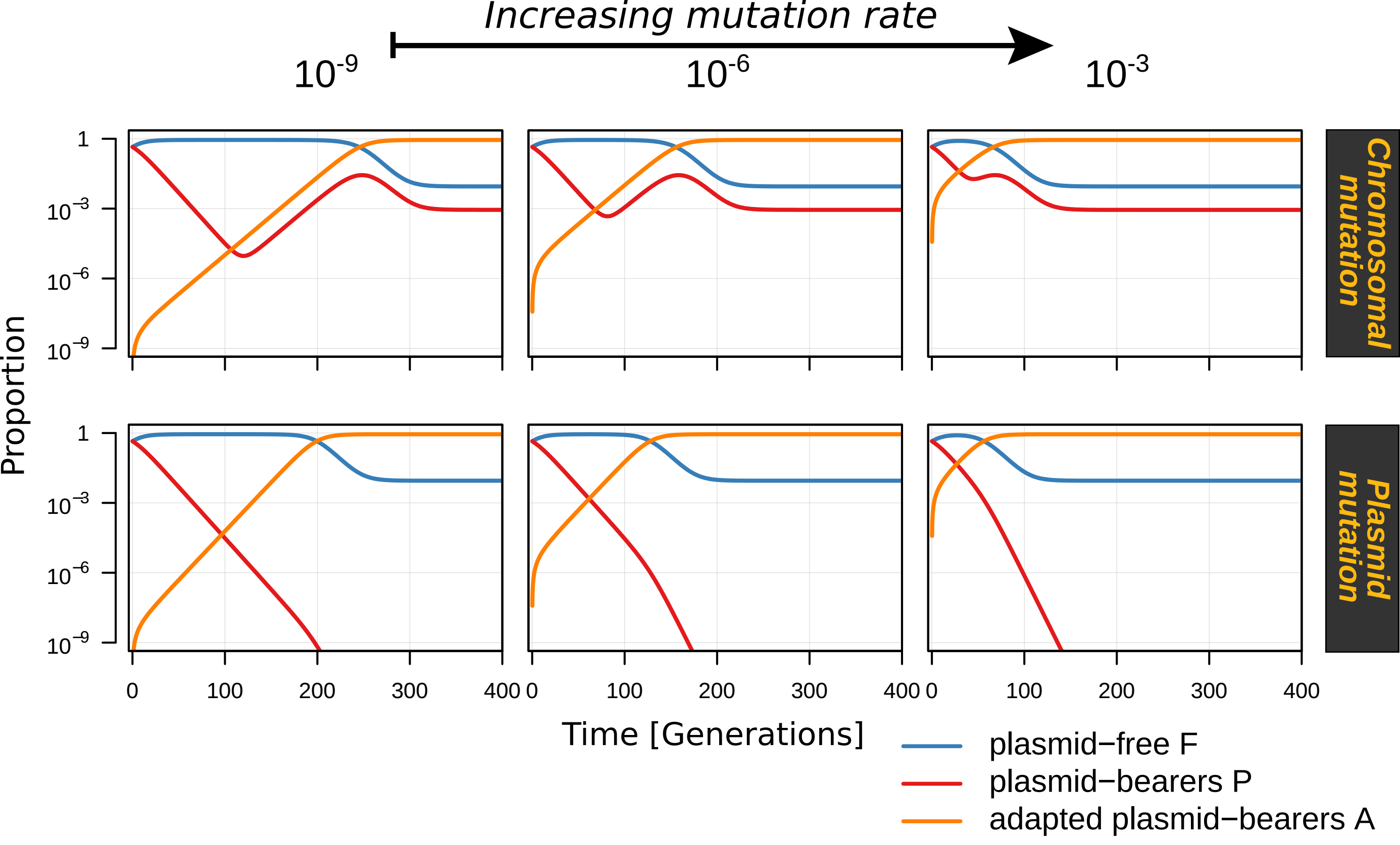

Supplement: FIG S3 [file sys001192313sf3.tif]

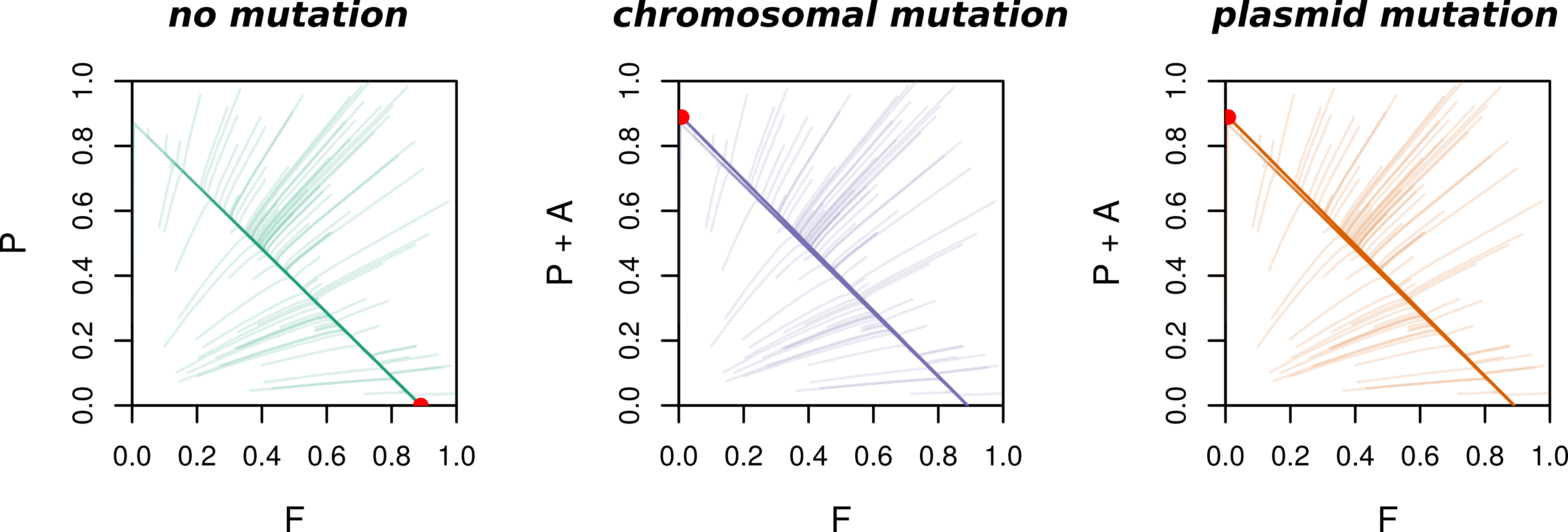

Supplement: FIG S4 [file sys001192313sf4.tif]
